# Supplementary material for: Elevated UTI Biomarkers in Symptomatic Patients with Urine Microbial Densities of 10,000 CFU/mL Indicate a Lower Threshold for Diagnosing UTIs
Source: Diagnostics (Basel). 2023 Aug 16;13(16):2688. doi: 10.3390/diagnostics13162688 (PMC10453813; doi:10.3390/diagnostics13162688)
Supplement: Supplementary file 1 [file diagnostics-13-02688-s001.zip › diagnostics-2503151-supplementary-.pdf]

Supplementary Table S1. Inclusion and Exclusion Criteria.

Inclusion	Exclusion
At least 60 years of age	
Male or Female sex<br>(no predetermined quotas or ratios for participation)	
Presenting to a urologist or urogynecologist in an outpatient setting	
Requires microbial testing according to clinician judgment	Failure to meet all inclusion criteria
Sample ordered with an ICD-10-CM code associated with UTI	
Sample contained enough urine to conduct M-PCR/P-AST, SUC, and biomarker assays on the same sample	

Supplementary Table S2. Bacterial Identification and Frequency by M-PCR and SUC in UTI symptomatic patients.

Organism	Method	Cases	%		Organism	Method	Cases	%	
E. coli	*SUC*	160	27%	Gram-negative	*E. faecalis*	*SUC*	150	26%	Gram -positive
	M-PCR	188	32%			*M-PCR*	94	16%	
K. pneumoniae	*SUC*	59	10%		VGS	*SUC*	2	<1%	
	M-PCR	63	11%			*M-PCR*	84	14%	
P. mirabilis	*SUC*	29	5%		CoNS	*SUC*	32	5%	
	M-PCR	24	4%			*M-PCR*	25	4%	
P. aeruginosa	*SUC*	14	2%		*S. agalactiae*	*SUC*	9	2%	
	M-PCR	22	4%			*M-PCR*	12	2%	
Enterobacter Group	*SUC*	8	1%		*E. faecium*	*SUC*	7	1%	
	M-PCR	13	2%			*M-PCR*	10	2%	
M. morganii	*SUC*	4	1%		*S. aureus*	*SUC*	3	1%	
	M-PCR	9	2%			*M-PCR*	5	1%	
C. freundi	*SUC*	5	1%		*A. urinae*	*SUC*	4	1%	Fastidious
	M-PCR	6	1%			*M-PCR*	118	20%	
K. oxytoca	*SUC*	2	<1%		*A. schalii*	*SUC*	0	0%	
	M-PCR	4	1%			*M-PCR*	116	20%	
C. koseri	*SUC*	4	1%		*G. vaginalis*	*SUC*	0	0%	
	M-PCR	2	<1%			*M-PCR*	35	6%	
S. marcescens	*SUC*	1	<1%		*A. omnicolens*	*SUC*	0	0%	
	M-PCR	2	<1%			*M-PCR*	17	3%	
P. stuartii	*SUC*	0	0%		*U. urealyticum*	*SUC*	0	0%	
	M-PCR	1	<1%			*M-PCR*	5	1%	
A. baumannii	*SUC*	0	0%		*C. riegelii*	*SUC*	0	0%	
	M-PCR	0	0%			*M-PCR*	5	1%	
P. agglomerans	*SUC*	1	<1%		*M. hominis*	*SUC*	0	0%	
	M-PCR	0	0%			*M-PCR*	3	1%	
C. glabrata	*SUC*	1	<1%	Yeast	*C. parapsilosis*	*SUC*	0	0%	Yeast
	M-PCR	16	3%			*M-PCR*	1	<1%	
C. albicans	*SUC*	1	<1%		*C. auris*	*SUC*	0	0%	
	M-PCR	3	1%			*M-PCR*	0	0%	
